# Empirics in Austria

**Published:** 1896-06

**Authors:** 


Empirics in Austria. At a session of the Austrian Landtag, December 18, 1895, in discussing a proposition of the Minister of the Interior, representative Dr. Ebenbroch spoke of the great need of veterinary surgeons in the country districts, and opposed the report of the Committee on Veterinary Epidemics, which recommended the education of veterinarians, who should first pass through an intermediate school and then study four years at a veterinary high school. He asserted that the agricultural conditions need cheap, practical veterinarians who are thoroughly conversant with the conditions in the country. Whether they are masters of the theories and the medical classics is a matter of indifference. The admission of the blacksmiths who had been employed in the army to veterinary practice was to be especially recommended. As to the agreement with Germany in regard to epidemics, he maintained that the division of Austria into two epidemic districts should have been made long ago. He then made complaint about the laws respecting epidemics, which, he maintained, had been enforced with too great severity.

Freiherr von Erb, representative of the government, remarked, in regard to the need of veterinarians, that the abuse of the laws prescribing the punishment of empirics had ceased, that a certain group of veterinarians who stand between those with a diploma and the empirics are useful to a certain extent. The ministry had already made the suggestion that the elements of veterinary medicine should be taught in all the agricultural schools. But to make such half-educated people the equals of veterinary surgeons with diplomas was impossible. The use of severe measures in carrying out the epidemic laws had not been necessary in Austria in recent years. Severe quarantine regulations in Salzburg had been necessary, because the presence of epidemics there had several times been kept secret.—*Ibid.*

